# Revealing the mechanisms of semantic satiation with deep learning models

**DOI:** 10.1038/s42003-024-06162-0

**Published:** 2024-04-22

**Authors:** Xinyu Zhang, Jing Lian, Zhaofei Yu, Huajin Tang, Dong Liang, Jizhao Liu, Jian K. Liu

**Affiliations:** 1 School of Information Science and Engineering, Lanzhou University, Lanzhou, 730000 Gansu China; 2 School of Electronics and Information Engineering, Lanzhou Jiaotong University, Lanzhou, 730070 Gansu China; 3 School of Computer Science, Peking University, Beijing, 100871 Beijing, China; 4 Institute for Artificial Intelligence, Peking University, Beijing, 100871 Beijing, China; 5 The State Key Lab of Brain-Machine Intelligence, Zhejiang University, Hangzhou, 310027 Zhejiang China; 6 The MOE Frontier Science Center for Brain Science and Brain-Machine Integration, Zhejiang University, Hangzhou, 310027 Zhejiang China; 7 Department of Computer Science and Technology, Nanjing University of Aeronautics and Astronautics, Nanjing, 211106 Jiangsu China; 8 School of Computer Science, Centre for Human Brain Health, University of Birmingham, Birmingham, B15 2TT UK

**Keywords:** Perception, Neural encoding, Visual system

## Abstract

The phenomenon of semantic satiation, which refers to the loss of meaning of a word or phrase after being repeated many times, is a well-known psychological phenomenon. However, the microscopic neural computational principles responsible for these mechanisms remain unknown. In this study, we use a deep learning model of continuous coupled neural networks to investigate the mechanism underlying semantic satiation and precisely describe this process with neuronal components. Our results suggest that, from a mesoscopic perspective, semantic satiation may be a bottom-up process. Unlike existing macroscopic psychological studies that suggest that semantic satiation is a top-down process, our simulations use a similar experimental paradigm as classical psychology experiments and observe similar results. Satiation of semantic objectives, similar to the learning process of our network model used for object recognition, relies on continuous learning and switching between objects. The underlying neural coupling strengthens or weakens satiation. Taken together, both neural and network mechanisms play a role in controlling semantic satiation.

## Abstract (web summary)

This study, utilizing deep learning to model semantic saturation, shows its tie to repetitive visual stimuli processing by the primary visual cortex, suggesting a bottom-up neurocognitive process.

## Introduction

Have you ever engaged in prolonged contemplation of a linguistic entity to the extent that its semantic essence begins to elude you? Consider, for instance, the word “cat” Prolonged and unwavering fixation on this linguistic symbol may evoke an eerie sense of detachment. Abruptly, the very word “cat”, conventionally evoking imagery of endearing domesticated felines, appears to undergo a peculiar transmutation, losing its inherent signification, and even forfeiting its status as a recognizable linguistic construct. Iterative inscriptions or readings of “cat” yield an analogous outcome. The ceaseless recurrence of a single lexical unit or phrase can culminate in its ephemeral deprivation of semantic meaning, an intriguing psychological phenomenon referred to as “semantic satiation”^1–6^. It is imperative to note that these enigmatic encounters are not restricted solely to the domain of language but extend to encompass non-verbal entities that have been studied in both humans and animals with different experimental protocols and techniques^7–15^. With the more advanced methods recently developed, this phenomenon has been continuously investigated with newly identified biomarkers^16–23^.

In the realm of neuroscience, despite the invaluable advantages it offers, research in the field of semantic satiation has predominantly operated at a macro level, thus far constrained in its ability to establish pertinent links between macro-level satiation phenomena and the intricacies of micro-level neural activity^15,24^. Neurophysiological experiments, by necessity, entail the examination of neural responses under stimulus conditions during the deep sleep of animals to mitigate the influence of extraneous variables^25–27^. However, the contention arises that the assessment of semantic satiation should ideally transpire under conditions where animals are in an awakened state. This suggestion presents a conundrum when contemplating the integration of neuroscience experimental methodologies into the ambit of psychological research^28^. Consequently, while significant strides have been taken in deciphering the mechanistic underpinnings of semantic satiation, the intricate dynamics of this phenomenon continue to elude comprehensive understanding. This challenge stems from the innate susceptibility of participant performance to the nuances of experimental paradigms and tasks. Thus, the proposition of developing a mesoscopic model emerges as an attractive pathway for research endeavors, one that endeavors to bridge the chasm between micro-level neural activity and the macro-level manifestation of semantic satiation^29^.

In light of these challenges, here we endeavor to employ a novel approach, utilizing a continuous coupled neural network^30^ and a fully connected layer to construct an artificial neural network based on deep learning that simulates the cognitive mechanisms underpinning semantic satiation. The CCNN, inspired by the dynamics of primary visual cortex^31^, exhibits commensurate static and dynamic properties with real neurons. Previous research has demonstrated that, by appropriately configuring the parameters of the CCNN, model complexity can be reduced while faithfully replicating the electrophysiological signals of actual neurons^32–34^. Consequently, the CCNN holds the promise of facilitating large-scale simulation computations while mirroring the dynamical attributes of real neurons^35^. This approach is poised to facilitate efficient and accurate observations and quantification of experimental results pertaining to semantic satiation, thus advancing our comprehension of this enigmatic phenomenon.

Our findings serve to elucidate the temporal dynamics of the proposed ventral pathway model’s image classification accuracy using a deep learning approach, revealing a distinct pattern over time. Specifically, we observe an initial augmentation in classification accuracy as time progresses, reaching an apex, followed by a subsequent decline. This pattern of performance closely mirrors the phenomenon of semantic satiation, a well-documented phenomenon associated with fluctuations in human classification ability^36,37^. As a repeated stimulus persists, information processing within the primary visual cortex becomes increasingly enigmatic. This underscores the pivotal role played by the primary visual cortex in information processing and suggests a link between the occurrence of semantic satiation and the underlying mechanisms operating within this brain region. Consequently, our observations imply that semantic satiation may be characterized as a bottom-up process, with the primary visual cortex being a key player in this cognitive phenomenon.

## Results

### Semantic satiation as a neural network model via deep learning

**Fig. 1:**
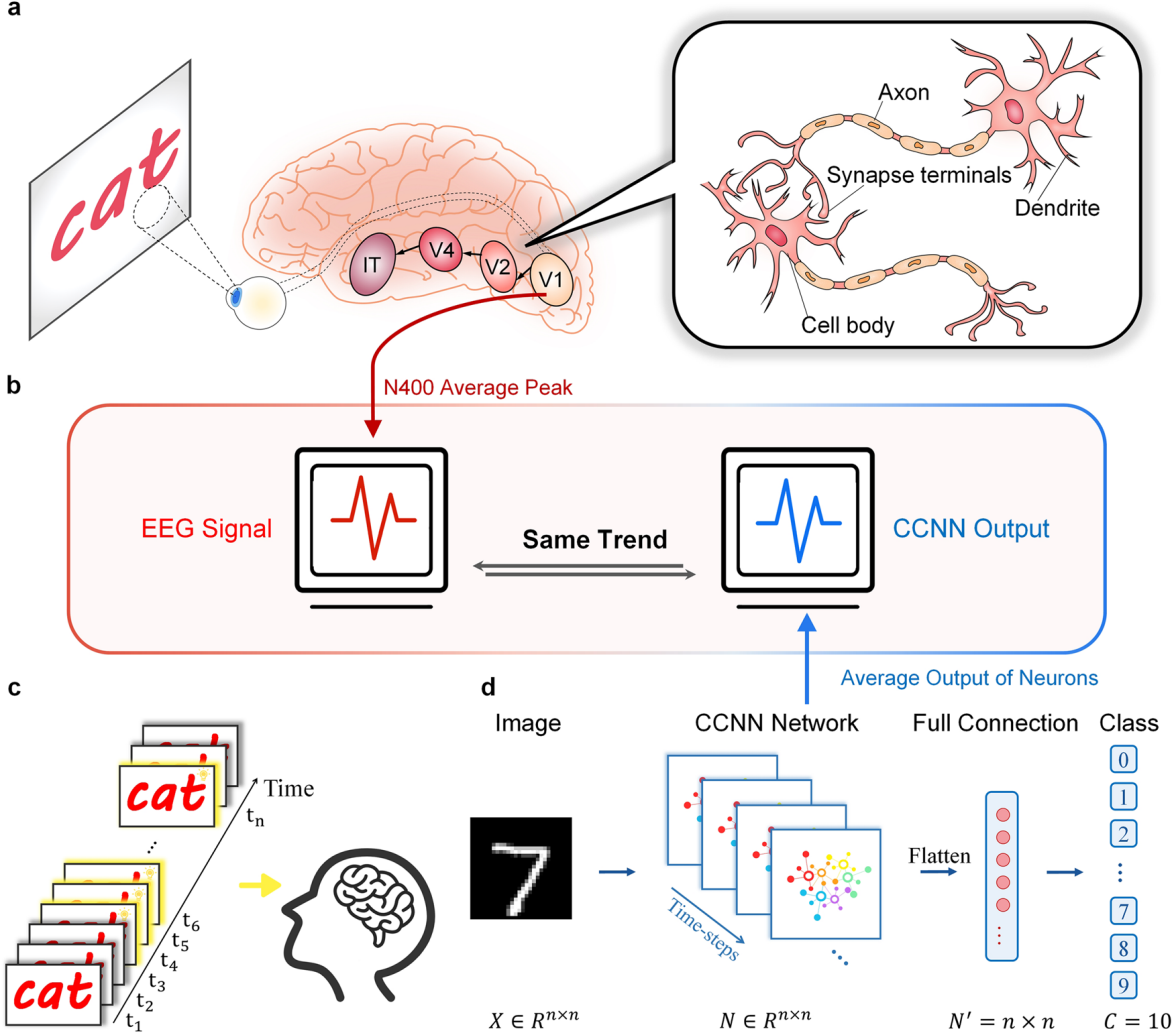
Comparison of ventral pathway and artificial ventral pathway framework. **a** The ventral visual pathway and neuronal connections in mammals. **b** Comparison of changing trends between our ANN primary visual cortex framework output and EEG signal. **c** Experimental paradigm of semantic satiation in psychology. **d** Artificial neural network semantic satiation framework based on CCNN.

Figure 1a provides a depiction of the veritable architecture of the ventral visual pathway within the visual cortex^38^. This neural pathway encompasses the sequential processing of light signals, commencing with their capture by the retina, followed by transmission through lateral geniculate nucleus (LGN) cells, further passage to the primary visual cortex (V1), and subsequent progression through secondary visual regions, including V2, V4, and the inferior temporal cortex (IT cortex)^39–43^. It is noteworthy that neurons located within V1 and V2 are characterized by smaller spatiotemporal integration receptive fields, allowing them to effectively process localized visual information^44,45^. In contrast, V4 neurons feature larger receptive fields, rendering them suitable for the integration of visual information within a broader visual field, while the IT cortex is primarily responsible for object recognition and higher-order cognitive functions.

Figure 1c portrays the semantic satiation paradigm in psychology, focusing on speed classification. This experimental design typically encompasses two critical processes: first, the repetitive presentation of identical stimuli to participants; and second, the participants’ subsequent judgments and responses, designed to gauge the impact of stimulus repetition on the accuracy of their classifications. In the realm of empirical studies on semantic satiation, a variety of experimental paradigms have been employed. Nonetheless, a common thread among these paradigms is the categorization task, which recurrently presents a target stimulus and requires participants to assess the category to which the target word belongs. Subsequently, the time required for categorization or the accuracy of judgment is assessed to explore the phenomenon. In the present study, we conceive of the primary visual cortex as a CCNN model that emulates the repetitive processing of input stimuli, with a fully connected layer simulating the recognition function of more advanced visual regions^46,47^. This model incorporates both inter-layer and intra-layer connections, mirroring the intricacies of the actual visual system, as portrayed in Fig. 1d.

The architecture and procedures of our network draw inspiration from classic semantic satiation experiments conducted by numerous psychologists. In this context, the accuracy of network-based classifications emerges as a pivotal metric for discerning the occurrence of semantic satiation. Furthermore, the average output of CCNN neural clusters serves as a representative indicator of the level of activity within the corresponding brain areas, facilitating comparisons with results from Electroencephalography (EEG) experiments^20–22^ as shown in Fig. 1b.

This proposed framework thus offers a relevant explanation for a multitude of intriguing findings in the domains of neuroscience and psychology, particularly those pertaining to semantic satiation. To evaluate the credibility of this model, we conduct a series of classification tasks that parallel psychology experiments. Our simulation framework, as delineated in Fig. 1c, endeavors to replicate various neuron behaviors observed in real-world experiments. Semantic satiation experiments in psychology, akin to the classification tasks in our study, involve the repetitive presentation of the same stimulus to participants^48^. Participants subsequently classify the presented stimulus after varying durations of exposure^49^, with the time required for classification serving as the determinant criterion for ascertaining the occurrence of semantic satiation.

As illustrated in Fig. 1d, our model framework is comprised of a network model, commensurate in size with the input image, designed to establish an Artificial Neural Network (ANN) simulating the primary visual cortex. The CCNN neurons are interlinked with neighboring neurons through linking matrices, with the dimensions of these matrices signifying the receptive field’s size. Subsequent to this initial stage, a fully connected layer, mirroring the functioning of the Inferior Temporal (IT) areas, is deployed to conduct the classification of the processing results generated by the ANN primary visual cortex. Our experimental protocol entails the repeated input of an identical image into the CCNN network layer for processing, with the number of iterations mirroring the stimulus presentation duration. The classification accuracy, a salient parameter, serves as a metric for appraising the network’s classification proficiency.

In the ensuing series of experiments, we employ two datasets, the MNIST dataset and the Fashion-MNIST dataset, to facilitate comprehensive simulations^50,51^. These datasets encompass both verbal and non-verbal images, affording us the capability to investigate the phenomenon of semantic satiation in both verbal and non-verbal contexts. The images serve as input stimuli, while the classification results constitute the model’s output. The number of repetitions corresponds to the duration during which primary visual cortex neurons process information and is consequently denoted as model time. This meticulous alignment with the experimental protocols in psychological studies enables a precise emulation of semantic satiation.

The primary objectives of the ensuing experiments are twofold: (1) To elucidate the emergence of visual-related semantic satiation at the mesoscopic level and ascertain whether it manifests as a bottom-up or top-down cognitive process. (2) To scrutinize the extent to which visual information undergoes modification within the primary visual cortex (V1) before reaching the inferior temporal cortex (IT) region, thus unraveling the intricate interplay between visual processing mechanisms and the phenomenon of semantic satiation.

### Semantic satiation caused by same repeated stimulus

Semantic satiation, a cognitive phenomenon, manifests when individuals are subjected to prolonged exposure to the same stimulus. Classic experiments in the realm of psychology have traditionally illuminated this phenomenon by repetitively presenting subjects with identical stimuli. The assessment of semantic satiation in such experiments typically hinges on quantifying the duration of stimulus presentation and the duration required for participants to reach a decision. Nonetheless, in conventional psychological experimentation, the precise quantification of participants’ response times in the context of varying stimulus presentation durations proves to be a formidable challenge. As a result, researchers often obtain only qualitative results, discerning whether the presentation time of the same stimulus is relatively shorter or longer. For instance, when the same target word is reiterated thirty times compared to three times, participants invariably exhibit extended decision-making times. In essence, excessive repetition of stimuli correlates with lengthened response times.

**Fig. 2:**
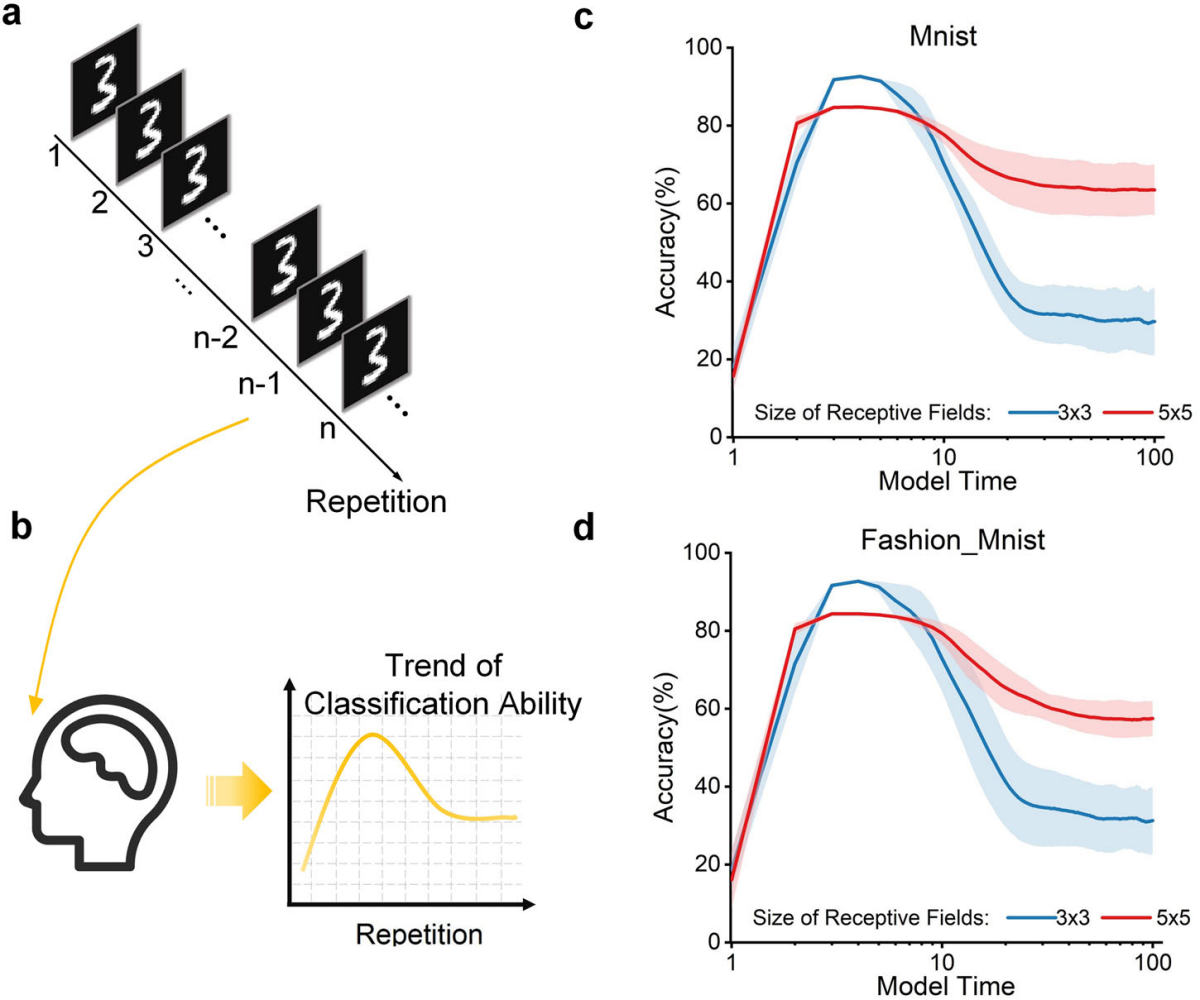
Semantic satiation caused by the same stimulus. **a** Input stimulus sequences in the experiment. The input stimulus remains the same during the model time process. **b** The trend of classification ability of participants in psychology experiments. The judgment ability of the participants first increases and then decreases as the model time increases. **c**, **d** The variation of MNIST and Fashion-MNIST classification accuracy with the model time under different receptive field sizes. The colored lines represent the size of receptive fields. The accuracy curve in various situations has the same trend as real psychological experiments. Receptive fields of different sizes show the same trend.

In our study, we emulate the experimental process in psychology, as delineated in Fig. 2a. The discernible trajectory of participants’ classification proficiency in psychological experiments is depicted in Fig. 2b. Our proposed Artificial Neural Network (ANN) framework is anchored in the same experimental paradigm. The results showcased in Fig. 2 indicate that the model’s accuracy initially ascends and subsequently declines with increasing model time. This trajectory mirrors the trends observed in psychological experiments (Fig. 2c, d). Notably, variations in the size of the receptive field do not fundamentally alter the overarching pattern. This underscores the efficacy of the ANN framework in faithfully simulating and reproducing the intricate process of semantic satiation. Significantly, the phenomenon of satiation is not confined solely to verbal stimuli but also extends to other non-verbal images, encompassing vision-related semantic satiation phenomena. This observation aligns seamlessly with the findings documented by psychologists.

### Semantic satiation caused by similar repeated stimulus

In classification tasks, showing a related word before the target word requires more time for the participant to make a classification decision^14^. For example, repeating “apple” thirty times before asking participants to classify “banana” or “chair” as fruit will significantly increase the time required to make a judgment for “banana” and result in more errors in classification. However, this effect does not occur when classifying “chair”. This suggests that stimuli related to the initial repeated stimulus are also affected by the satiation effect.

**Fig. 3:**
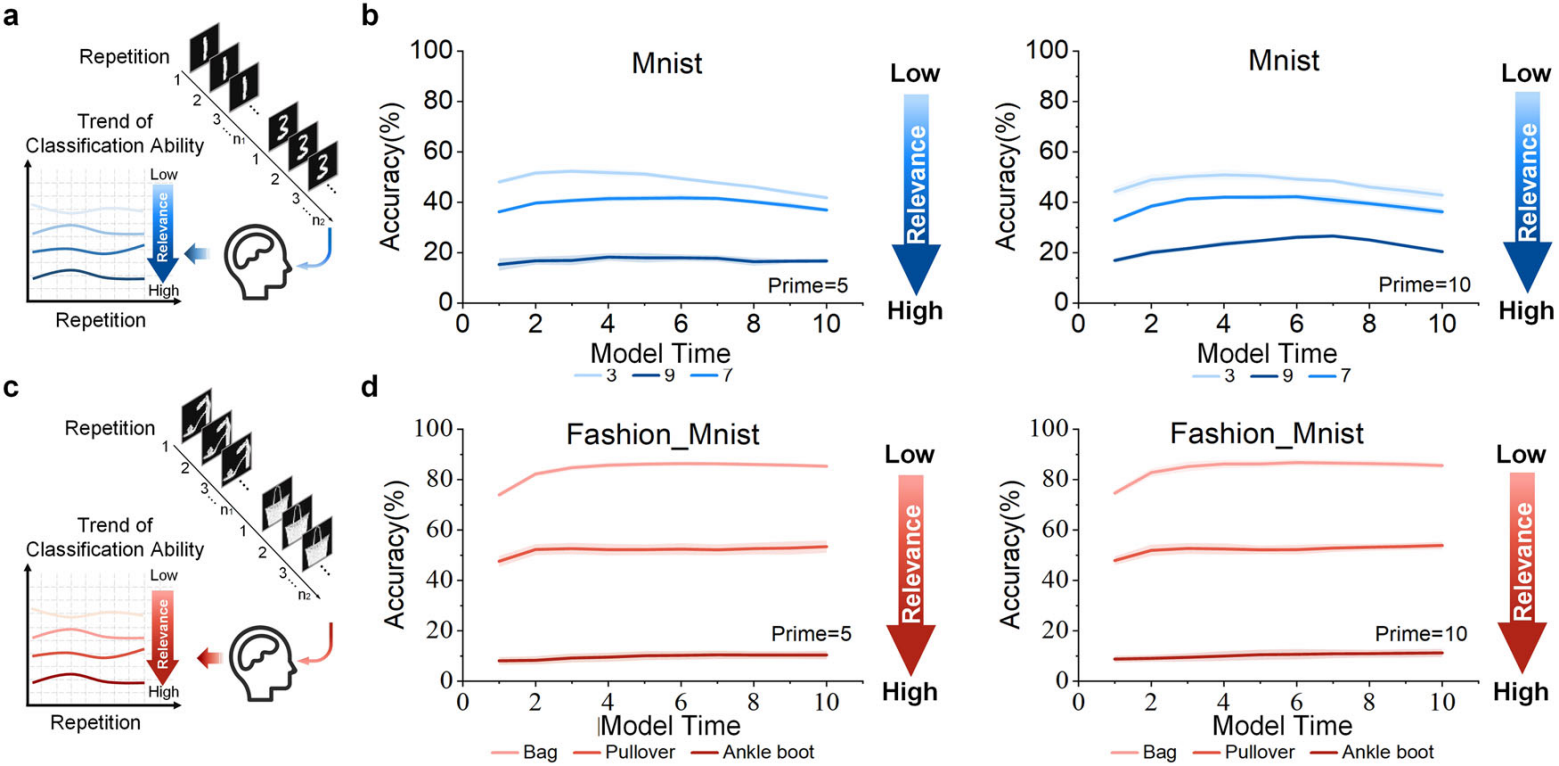
Semantic satiation caused by similar stimulus. **a**, **c** Similar input sequences from the MNIST and Fashion-MNIST datasets. The input is divided into two parts, starting with a stimulus input that is repeated 5 or 10 times before inputting the target stimulus. The participants show a higher classification ability of input-2 on the class with a lower relevance. **b**, **d** The trend of the accuracy of the target input (input-2). The left and right graphs show the cases where input-1 is repeated 5 and 10 times, respectively. The colored lines represent different categories of input-2. Darker colored lines represent higher category similarity and lower accuracy. The higher the similarity with input-1, the lower the accuracy of input-2. The results demonstrate that the proposed ANN framework is able to imitate the characteristics of semantic satiation in humans.

The aim of this experiment is to explore how neurons react to images with high relevance, low relevance, or relatedness after an extended period of stimulus. In section, Semantic satiation as a neural network model via deep learning, the size of the receptive field doesn’t have a significant impact on the experimental results. Therefore, in this experiment, the receptive field is set to 3 × 3. The experimental input sequence is shown in Fig. 3a, c. Firstly, a prime stimulus is input as input-1, and then after several repetitions of input-1, the target stimulus to be classified is input as input-2. By analyzing the correlation between input-2 and input-1 and the classification accuracy of input-2, the effect of the similarity between the different categories on the degree of satiation can be exposed through input-1 and input-2. Both datasets show the same phenomenon. There are different correlations between the different categories. For instance, some numbers may have higher relevance to the number “1” (e.g., “7”), while others may be less relevant (e.g., “9”), or unrelated (e.g., “3”). People tend to perceive more similarities between “1” and “7”, such as the shape of a straight line while finding a little resemblance between “1” and “3”. The Structural Similarity Index (SSIM) is used to measure the degree of similarity between different categories. In Fig. 3b, the SSIM value for “1-3” is 0.203, the SSIM value for “1-7” is 0.250, and the SSIM value for “1-9” is 0.243. In Fig. 3d, the SSIM value for “Sandal-Bag” is 0.045, the SSIM value for “Sandal-Pullover” is 0.067, and the SSIM value for “Sandal-Ankle boot” is 0.161. SSIM values between all categories in the two datasets are shown in Fig. S1. The subsequent study involves inputting one type of number image into the single-layer CCNN framework repeatedly and then inputting a different type of number image to measure the recognition accuracy of MNIST (Fig. 3a). The extent of the decrease varies according to the relevance of each number to input-1 as shown in Fig. 3b. The same results are obtained in the Fashion-MNIST dataset experiment as shown in Fig. 3c, d. Psychological study has shown that participants require more time to recognize the numbers “1” or “7”, as verified by EEG-based experiments^21^. Therefore, the results of this study are consistent with those of actual psychological experiments.

### Visualization of visual information processing’s intermediate state

**Fig. 4:**
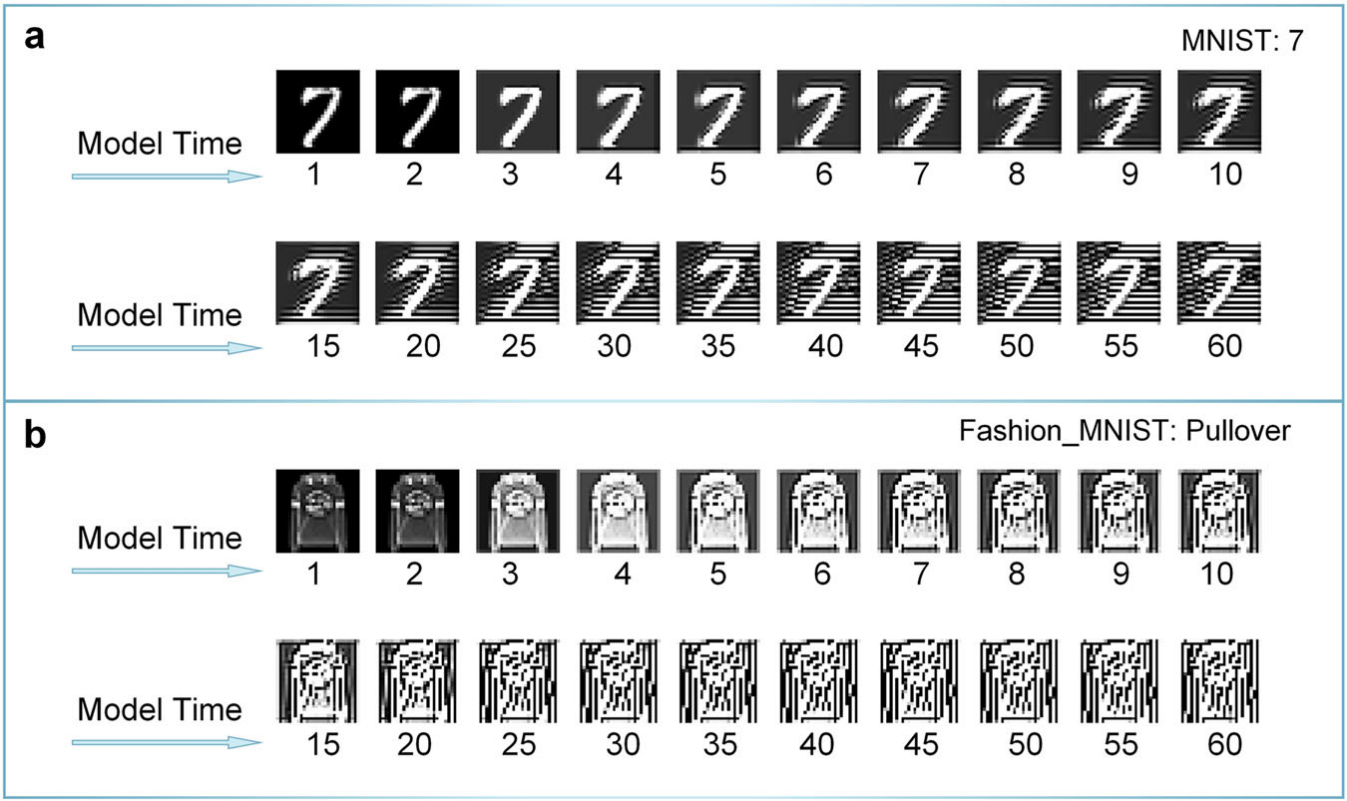
Visualization of the results of the processing of the CCNN at different time points. **a** Repeat processing results for number “7” in CCNN. **b** Repeat processing results for “pullover” in CCNN. Both datasets show that the images are changing as the model time lasts. This may make it more difficult to classify images for the model.

**Fig. 5:**
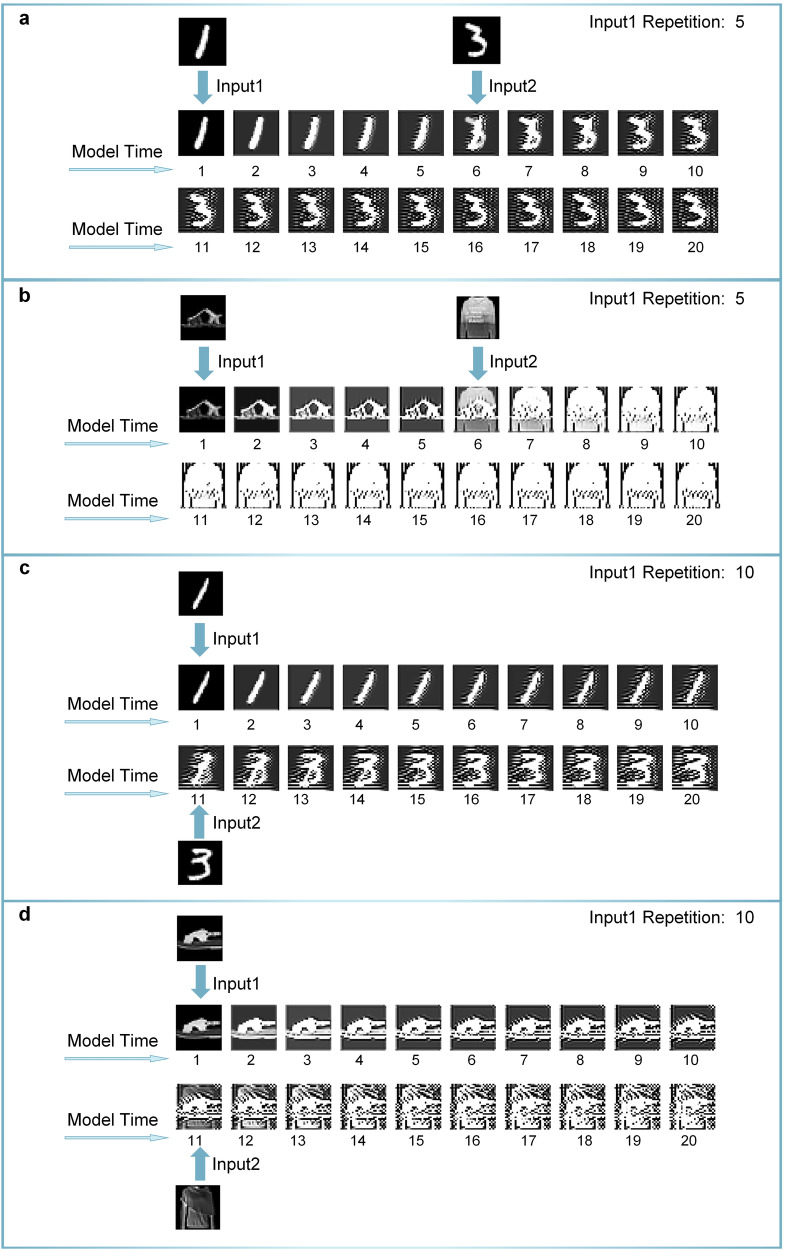
Visualization of semantic satiation caused by similar input. **a** The process of experiment on MNIST with the five repetitions of input-1. **b** The process of experiment on Fashion-MNIST with the repetitions of input-1. **c** The process of experiment on MNIST with the ten repetitions of input-1. **d** The process of experiment on Fashion-MNIST with the ten repetitions of input-1. In fig. (**a**, **c**), “1” repeats 10 times has a greater impact on the following images than 5 times. The same result can be seen on Fashion-MNIST in fig. (**b**, **d**). Continuous repetition will make it more difficult for the model to classify similar content.

The experiments above demonstrate that the model is capable of simulating semantic satiation from multiple perspectives. However, the evolution of semantic or visual signals over time remains unclear. The output feature of the CCNN layer at individual time points and the outputs of the fully connected layer are plotted to study the evolution of semantics in the primary visual cortex and IT area. The visualization includes the following parts:

Figures 4 and 5 show the changes in the image in our ANN primary visual cortex. Figure 4 displays the images processed by the ANN network at various model times, corresponding to section, Semantic satiation as a neural network model via deep learning. In both datasets, the image signals gradually become more obscure and the main parts, which refer to the parts related to semantics, become larger and more unclear (Fig. 4a, b). Figure 5 shows the changes in a similar stimulus experiment, corresponding to section, Semantic satiation caused by same repeated stimulus. The effect of continuous pre-stimulus persists for some time after the stimulus stops. The accuracy of other input1 and input2 situations can be found in Fig. S2. The residual image of the processing result of the first image signal appears on the processing result of the other subsequent pictures for some time before gradually fading away.

Owing to the existence of coupling connections, the firing information of neurons propagates through the coupling links to neighboring neurons, giving rise to an automatic wave effect. As model time progresses, this automatic wave effect becomes susceptible to interference from high-intensity waves present in the darker regions of the image. Consequently, this interference disrupts the state of neurons in the darker areas and distorts the features at the image’s edges. The temporal evolution of several intermediate states of the CCNN neuron was visualized in Fig. S3. The five main parts are feeding input F, couple linking L, modulation product U, dynamic activity E, and continuous output Y. Due to the presence of coupling connections, noise gradually spreads towards the surrounding dark areas.

**Fig. 6:**
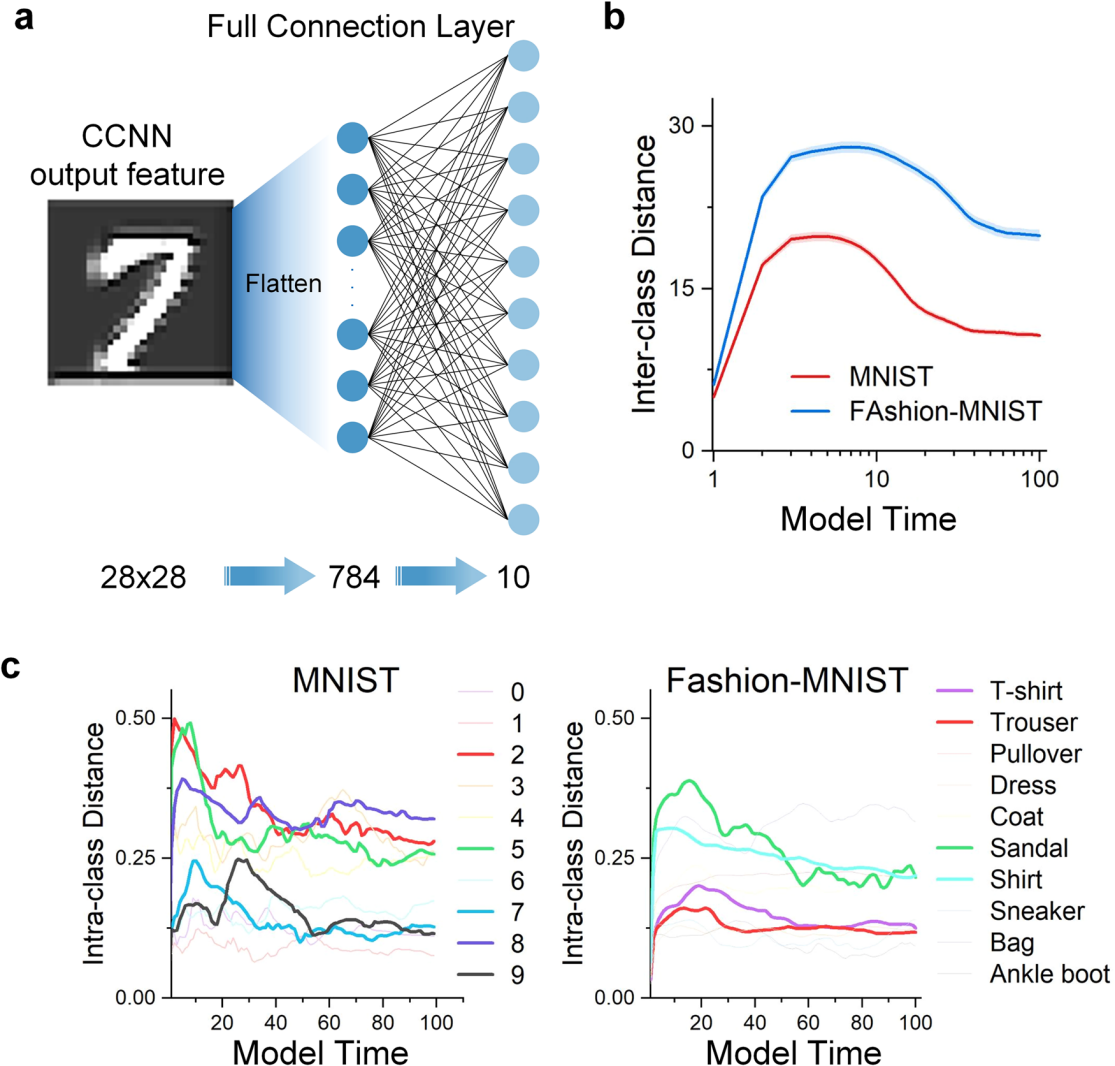
Intra-class and inter-class distance of semantic vectors. **a** The details of the fully connected layer. This layer extracts 784 elements into a 10-dimension vector. **b** The inter-class distance of MNIST and Fashion-MNIST datasets. **c** The intra-class distance of MNIST and Fashion-MNIST datasets. In both datasets, the inter-class distance shows a trend of first rising and then falling. From the results, for the model, the larger the inter-class distance, the easier the classification will be. However, the intra-class distance shows a more complicated situation.

**Fig. 7:**
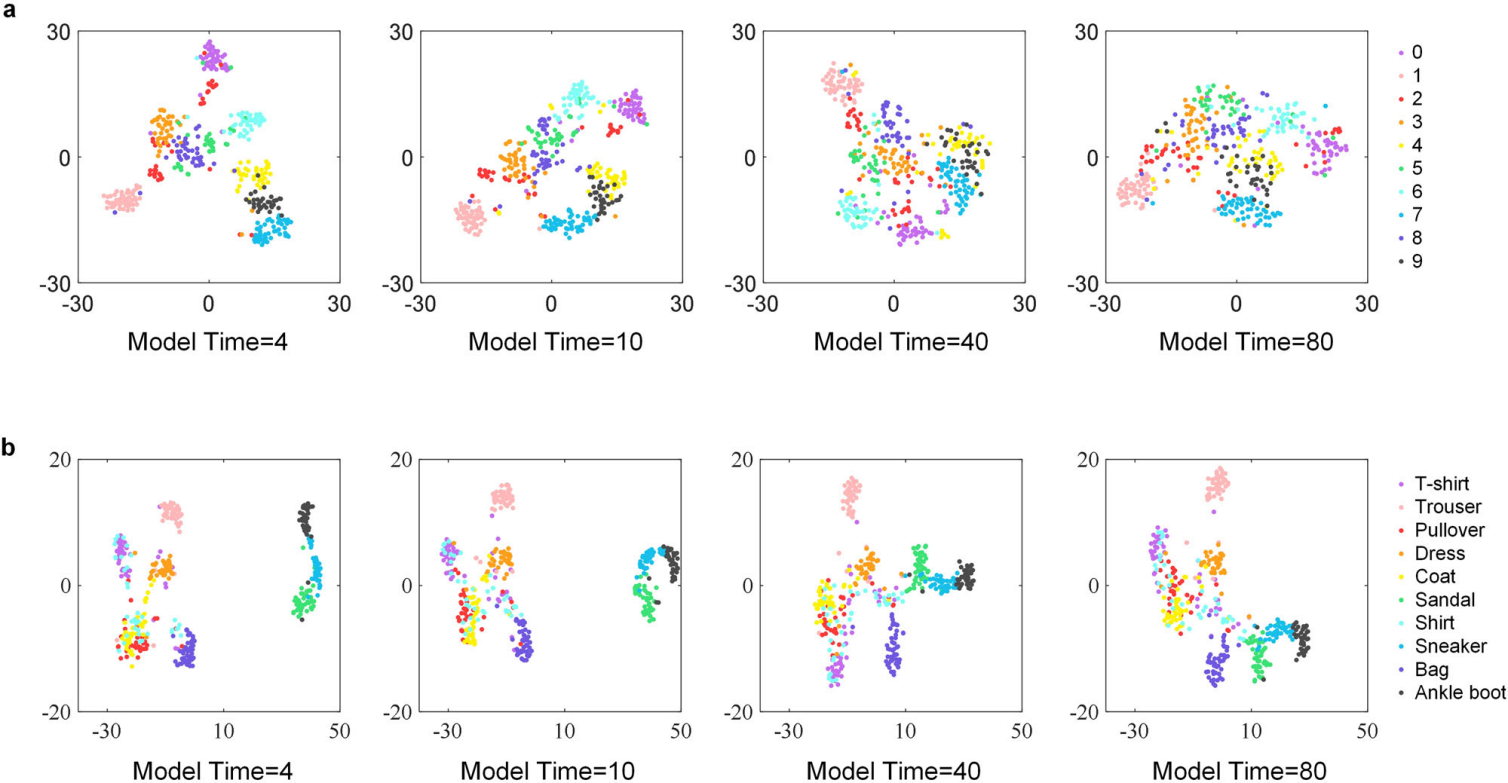
Semantic distance for different categories. **a** Two-dimensional vectors of the semantics of MNIST. **b** Two-dimensional vectors of the semantics of Fashion-MNIST. The distribution of semantics shows a trend of being gradually disordered.

Figures 6 and 7 show the variation in processing results for the manual IT area. The fully connected layer abstracts each image into a ten-dimensional vector in Fig. 6a. Then, the distance between the mean values of different categories of vectors can be calculated. This simulation uses abstract digital vectors to represent semantic segments. The inter-class distance’s variation trend is consistent with the accuracy, but the intra-class distance’s trend does not move in the opposite direction as expected as shown in Fig. 6b, c^52^. Figure 7 shows the vector distribution after t-distributed stochastic neighbor embedding (t-SNE). This indicates that in the semantic satiation phenomenon, the semantics of different classes become more dispersed, making classification more challenging to complete and even leading to errors.

In summary, semantic satiation is closely related to the working mechanisms of the primary visual cortex. Due to the connection between neurons, the continuous stimulus of a single neuron will impact the action potential and signal transmission process of other surrounding neurons, which consequently alters the information processed by all neurons. This explains why the persistent input signal to the brain may lead to the attenuation of definition. From Fig. 5, it can be inferred that neuronal responses exhibit a certain time delay compared to stimulus signal input. In Fig. 5, the residual image of the previous input signal is still retained in the output even after the signal has ceased for a while. This delay prevents neurons from swiftly returning to the initial state and adapting promptly to new tasks. And it is able to explain well the changes in the semantic distribution of the IT area.

### Comparison experiment of framework and EEG based cognitive study

**Fig. 8:**
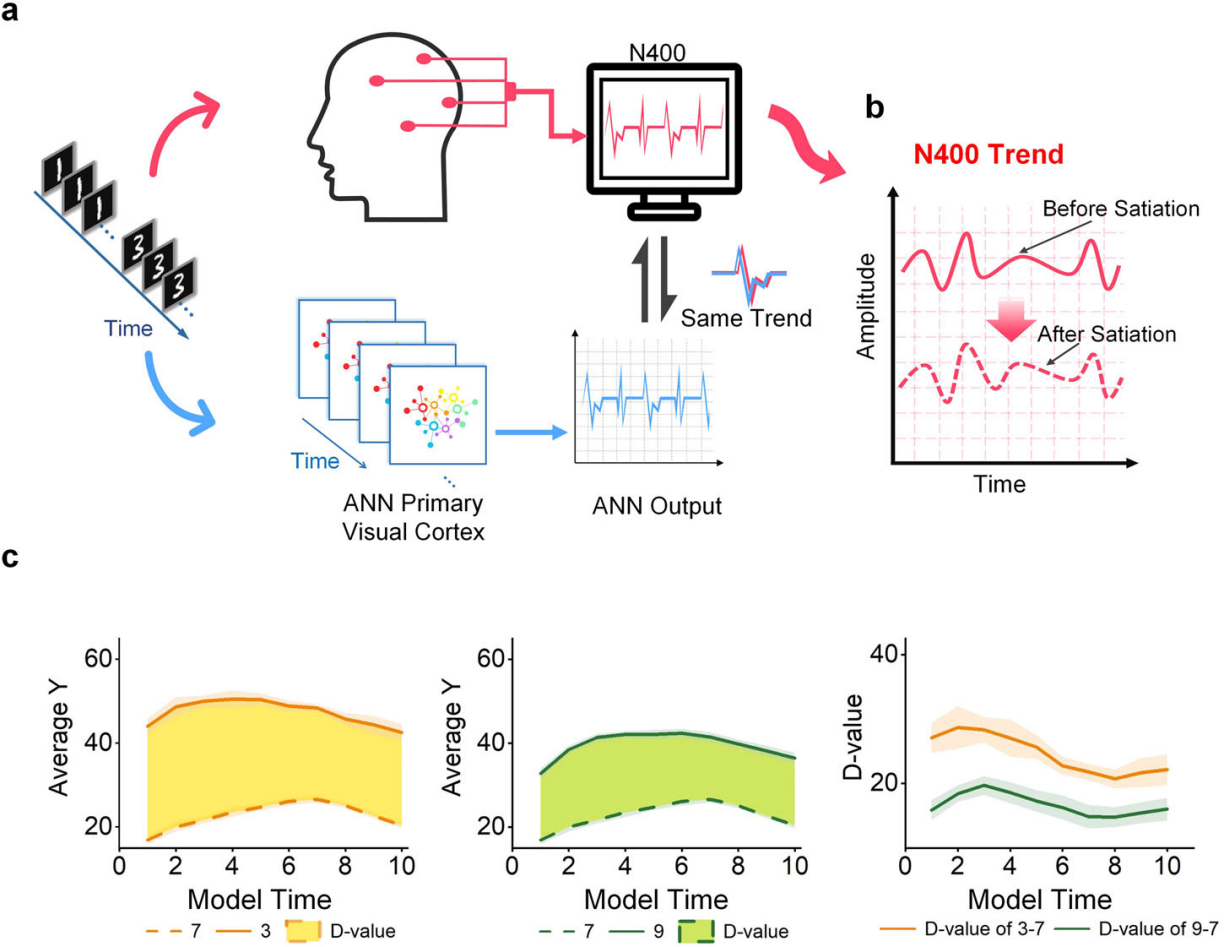
Comparison with the trend of N400 composition. **a** Schematic diagram of the experimental process in psychology and ANN model. The change of them has the same trend. **b** The changing trend of N400 before and after satiation. Semantic satiation makes the N400’s Amplitude become lower. **c** Trend of the average output (*Y*) difference (D-value) of different categories. The left plot shows the average output of “3” and “7”. The middle plot shows the average output of “9” and “7”. The right one shows the *D*-value of the output *Y*: “3 − 7” and “9 − 7”.

Psychological perspectives have utilized the N400 component of event-related potentials (ERPs) to investigate the phenomenon of semantic satiation. Researchers observe the amplitude changes of the N400 component before and after semantic satiation^20,21,53^. When two completely unrelated stimuli are presented, significant differences in the amplitude of the N400 component will occur. The difference in N400 of population activities before and after satiation can be used to measure the degree of satiation as shown in Fig. 8b^54–57^. When the difference is larger, the degree of satiation is generally believed to be deeper.

Parallels have been drawn between the neuronal action potentials generated by the CCNN model and the N400 component observed in EEG signals due to their shared manifestation as electrical signal characteristics indicative of neural activity. EEG signals serve as a proxy for neuronal activity fluctuations transmitted by electrical signals across the brain. Similarly, the output Y from the CCNN model’s neurons, representing action potentials, reflects the underlying neuronal activities. N400 typically manifests itself in the central and upper regions of the brain^58^. For a comprehensive understanding of the spatial distribution of the N400 effect and the interaction between brain regions, multiple electrodes are typically employed for recording^59^. This facilitates the analysis of N400 waveform distribution across the entire scalp and the exploration of spatiotemporal relationships between different brain regions. Drawing from psychological experiments on N400, researchers primarily focus on changes in N400 amplitudes before and after semantic satiation^53^. When two entirely unrelated stimuli are presented, significant differences in N400 component amplitudes arise. The disparity in the amplitudes of the N400 components before and after satiation serves as a measure of the degree of satiation^54^.

In our model, the average output of neurons in the ANN primary visual cortex represents the level of neuron activity. By comparing the output difference of the visual cortex before and after semantic satiation, it can be determined whether there is a satiation phenomenon in this stage. As shown in Fig. 8a, the output of our model has the same trend as the change in N400. Our experiment yielded a similar conclusion: once semantic satiation sets in, the difference in the mean output of the neurons significantly reduces. The experiments compared the average output of our model’s neurons at input-2 from the experiments in Section, Semantic satiation caused by same repeated stimulus, as shown in Fig. 8c. The difference in output between the numbers “7” and “3” is significantly higher than the difference in output between the numbers “7” and “9”. The disparity gradually decreased following the onset of semantic satiation. Our findings resemble those of N400 experiments conducted by cognitive psychologists, however, our conclusions differ. Our experimental results show that semantic satiation emerges in the primary visual cortex. This difference may be attributed to the spatial scale of our respective investigations. While existing cognitive research based on EEG characterizes cognitive processes by employing the average activity of numerous neurons in the brain region, our research examines cognitive processes using mesoscopic neural networks. Hence, this study presents a different landscape.

## Discussion

Early studies in the field of psychology coined the term “Semantic Satiation”^1^ to delineate the phenomenon wherein the repetition of a stimulus or context precipitates a waning of the affective response. The conceptual underpinning of “satiation” first materialized in the context of a comprehensive meta-analysis of research endeavors related to maze-running experiments in the context of rodent behavior^7^. Divergent from response inhibition, “satiation” pertains to a form of aversion related to the continued processing of a recurrent stimulus or context, specifically denominated as “processing satiation”^8^. The exploration of semantic satiation has piqued the interest of numerous psychologists, engendering a proliferation of research methodologies, thereby yielding a kaleidoscope of distinctive theories^1,3,4^. However, hitherto, the collective scientific endeavor has not succeeded in providing a definitive elucidation concerning the precise neural mechanisms and locales implicated in the manifestation of this intriguing phenomenon.

The inquiry into the phenomenon of semantic satiation has embarked upon a protracted and intricate journey, marked by methodological evolution and refinement^9^. In its nascent stages, research predominantly leaned on introspective methods, which necessitated participants to expound upon their own cognitive experiences, encompassing thoughts, emotions, and consciousness^10–12^. Of notable significance, Lambert’s seminal investigation entailed participants evaluating their affective responses to repeatedly presented words^1^. The findings of this study revealed a perceptible attenuation in participants’ emotional responses following the repetition of identical words, thus manifesting a marked semantic erosion. However, by employing a closely analogous experimental approach, a congruent phenomenon was intriguingly failed to observe^13^. The inherent limitation of introspective methods lies in their challenge in quantifying and standardizing participants’ subjective experiences^14^, thus yielding inconsistent findings that have hindered the advancement of semantic satiation research. Consequently, introspective methods prove insufficient for discerning the intricate stages underpinning the emergence of semantic satiation^15^.

Subsequent to this nascent phase, the burgeoning fields of cognitive psychology and cognitive neuroscience ushered in a plethora of diverse experimental paradigms and neurophysiological techniques, marking a new era of objective and quantifiable approaches to investigating semantic satiation^16–19^. Notably, the N400 component emerged as a prominent tool in probing the processes underlying semantic satiation^20–22^. This component is commonly employed to measure semantic processing, with greater amplitude observed in cases of semantic or contextual incongruence compared to congruence. While experimental outcomes have not been universally consistent, they collectively support the conclusion that semantic satiation transpires at the semantic level, rather than being a mere offshoot of perceptual decay. Notably, magnetoencephalography (MEG) was used to discern that satiation materializes at the juncture where perception and semantics converge^23^. Not with standing the advantages afforded by neuroscience, research in this domain has primarily operated at a macro level, with a limited capacity to establish links between macro satiation phenomena and micro-level neural activity. Neurophysiological experiments necessitate the examination of neural responses under stimulus conditions during the deep sleep of animals to mitigate extraneous interference. However, it is suggested that the evaluation of semantic satiation should be conducted under conditions where animals are awake, presenting a potential challenge when integrating neuroscience experimental methodologies into psychological research.

Therefore, while several strides have been made in elucidating the mechanism of semantic satiation, the intricacies of the phenomenon continue to elude complete understanding due to the susceptibility of participant performance to the intricacies of experimental paradigms and tasks. Hence, the development of a mesoscopic model that bridges micro-level neural activity with the macro-level manifestation of semantic satiation appears to be a promising avenue. In this study, drawing from the intricate physiological underpinnings of neurons within the visual cortex, we have engineered an ANN semantic satiation framework rooted in the CCNN. This innovative framework endeavors to improve the inherent limitations of real-life psychological experiments, which are susceptible to disruptions and possess complexities that challenge their reliability. Our empirical findings resonate with previous studies, conclusively establishing that semantic satiation transpires upon the recurrent presentation of identical or closely akin stimuli. Yet, our mesoscopic-scale investigation introduces an intriguing perspective, postulating that semantic satiation could be characterized as a bottom-up cognitive process. This novel perspective holds the potential to furnish the realm of psychological research with numerical benchmarks and methodological paradigms. However, owing to the constraints of our present experimental conditions, we are currently unable to validate our simulation results on actual neurons. Nevertheless, our research proffers novel insights and avenues for future exploration, thereby extending an invitation for subsequent research endeavors to substantiate our discoveries.

In particular, these implications appear to diverge from the prevalent psychological research, which generally posits semantic satiation as a top-down process^22,60,61^. Although our study aligns with psychological research in its choice of experimental paradigm, discrepant views may be attributable to discrepancies in the scales of observation. It is imperative to underscore that our experimental results are underpinned by careful examination, and we note a congruence between the overall neural output levels in the CCNN model and the observed trends in the N400 signal as documented in classical experiments. Both the N400 components and the CCNN model’s output exhibit a discernible decline in the context of semantic satiation prompted by heightened image similarity. Furthermore, our real-time monitoring of individual neuron activity unveils a distinct neural landscape, potentially affording novel insights and a mesoscopic perspective for the continued exploration of the intricate phenomenon of semantic satiation. This holistic approach, bridging computational modeling and neural activity analysis, provides a promising avenue for further unraveling the cognitive intricacies of this phenomenon. Notably, in psychological experiments, the process typically involves two main stages: presenting prime words (input-1) and target words (input-2), with a deliberate pause, referred to as a “blank wait” period, between each stimulus presentation^53^. This pause is crucial for ensuring that participants’ attention remains squarely focused on the visual stimuli. In our model, unlike the human brain, there isn’t an element of uncontrolled distraction; consequently, the “blank wait” phase was not initially incorporated. Therefore, integrating distraction and attentional focus in future research could significantly enrich our understanding and the realism of our model.

Moreover, our model proposes broader inquiries that may be interesting in the domain of psychological experimental research. What are the advantageous aspects of this operational mechanism within the human brain? Can it be deemed the optimal mode of information processing in the cerebral apparatus, and how might we harness or circumvent this phenomenon? These intriguing questions, grounded in our computer simulation outcomes, eagerly await corroboration through dedicated biological and psychological experiments. It is possible that these information-processing mechanisms could serve as sources of inspiration for brain-inspired computing. In general, our results provide the potential to study semantic satiation using neural network models, which may spark the emergence of novel research questions that span the domains of brain-inspired computing and psychology.

## Methods

In this study, all simulations employ a uniform model structure and utilize the MNIST and Fashion-MNIST datasets as inputs. Each experiment adopts different input, analysis, and statistical techniques. The architecture mainly comprises two components: the ANN primary visual cortex layer and the artificial IT layer, as illustrated in Fig. 1. The following parts will outline the principles of the two layers and explicate the specific stages and intricacies of network training and testing.

### The ANN primary visual cortex layer

The ANN primary visual cortex layer is composed of the CCNN network. In the realm of modeling the visual pathway, Convolutional Neural Networks (CNNs) have been widely adopted due to their efficacy in emulating the functioning of the visual cortex. However, a notable limitation of traditional CNNs is their lack of capacity to incorporate temporal dynamics, synchronous oscillations, and refractory periods, which are intrinsic to the behavior of real neurons. This disparity prompted exploration into alternative models offering a closer approximation to the physiological processes of the human visual system. The Pulse-Coupled Neural Network (PCNN), inspired by the groundbreaking work of Eckhorn^62^ and further elaborated by Rangnanath^63^, is a model with high biological rationality. PCNNs, with their feedback mechanism and spike coding, provide a more physiologically aligned model. The equations of PCNN are as follows:

1 $$\left\{\begin{array}{l}{F}_{ij}(n)={e}^{-{\alpha }_{f}}{F}_{ij}(n-1)+{V}_{F}{M}_{ijkl}{Y}_{kl}(n-1)+{S}_{ij}\quad \\ {L}_{ij}(n)={e}^{-{\alpha }_{l}}{L}_{ij}(n-1)+{V}_{L}{W}_{ijkl}{Y}_{kl}(n-1)\hfill \quad \\ {U}_{ij}(n)={F}_{ij}(n)(1+\beta {L}_{ij}(n))\hfill \quad \\ {Y}_{ij}(n)=\left\{\begin{array}{ll}1,\quad &if\quad {U}_{ij}(n)\, > \,{E}_{ij}(n)\\ 0,\quad &otherwise\end{array}\right.\hfill \quad \\ {E}_{ij}(n)={e}^{-{\alpha }_{e}}{E}_{ij}(n-1)+{V}_{E}{Y}_{ij}(n-1)\quad \hfill \end{array}\right.$$

Where (*i*, *j*) is the (*i*_*th*_, *j*_*th*_) neuron, and (*k*, *l*) is the (*k*_*th*_, *l*_*th*_) neuron. The five main parts are couple linking *L*_*ij*_(*n*), feeding input *F*_*ij*_(*n*), modulation product *U*_*ij*_(*n*), dynamic activity *E*_*ij*_(*n*) and output *Y*_*ij*_(*n*). *S*_*ij*_ represents the external feeding input obtained by the receptive fields. *α*_*f*_, *α*_*l*_ and *α*_*e*_ are exponential decay factors. *V*_*F*_ and *V*_*L*_ denote weighting factors. Moreover, *W*_*ijkl*_ and *M*_*ijkl*_ indicate feeding and linking synaptic weights, respectively, and *β* indicates the linking strength, which directly determines *L*_*ij*_(*n*) in the modulation product *U*_*ij*_(*n*). *S*_*ij*_ is the external feeding input, *α*_*f*_, *α*_*l*_, *α*_*e*_ are exponential decay factors of the five main parts.

However, a critical gap in the PCNN model’s capacity to replicate the dynamic responses of neurons to external periodic signals was identified by Siegel’s experiments^64^ with real primary visual cortex neurons. This limitation motivated the development of the (CCNN)^30^, which was introduced to address the shortcomings of the PCNN in capturing the complex nonlinear dynamics of neuron activity under dynamic stimuli. The CCNN comprises five components: coupling linkage, feeding input, modulation product, dynamic activity, and continuous output. The CCNN equations are as follows:

2 $$\left\{\begin{array}{l}{F}_{ij}(n)={e}^{-{\alpha }_{f}}{F}_{ij}(n-1)+{V}_{F}{M}_{ijkl}{Y}_{kl}(n-1)+{S}_{ij}\quad \\ {L}_{ij}(n)={e}^{-{\alpha }_{l}}{L}_{ij}(n-1)+{V}_{L}{W}_{ijkl}{Y}_{kl}(n-1)\hfill \quad \\ {U}_{ij}(n)={F}_{ij}(n)(1+\beta {L}_{ij}(n))\hfill \quad \\ {Y}_{ij}(n)=\frac{1}{1+{e}^{-({U}_{ij}(n)-{E}_{ij}(n))}}\hfill \quad \\ {E}_{ij}(n)={e}^{-{\alpha }_{e}}{E}_{ij}(n-1)+{V}_{E}{Y}_{ij}(n-1)\hfill \quad \end{array}\right.$$

Where, the output *Y*_*ij*_(*n*) is changed from a pulse signal to a continuous value. The CCNN model distinguishes itself by generating continuous output values, *Y*_*ij*_(*n*), and demonstrates behavior that closely mirrors the dynamic of chaotic activity observed in real V1 neurons when exposed to periodic stimuli. This adaptation enables the CCNN to better simulate the intricate dynamics of neuron interactions within the primary visual cortex, offering a model that strikes an optimal balance between biological fidelity and the complexity of neuronal behavior.

In experiments, all neurons are in a static state at the beginning, then all parameters in the matrices *F*, *L*, *U*, *Y* and *E* are zero in the initial state. The parameter settings in all experiments are as follows: *α*_*f*_ = 0.1, *α*_*e*_ = 1, *α*_*l*_ = 0.1, and *β* = 0.5. The impact analysis of these parameter settings on the model is detailed in Fig. S4(see Supplementary Figures).

The ANN model of the primary visual cortex encompasses neurons of identical size as the input images, totaling 784 (28 × 28) neurons. The size of the receptive field is set to 3 × 3 and 5 × 5 for simulation experiments. In the specific computation, we realize this calculation by convolution without bias.

### The ANN model of IT area

This model’s IT area employs the fully connected layer to reduce the dimensionality and classify the information processed by CCNN as shown in Fig. 6a^65,66^. Following the iterative processing of CCNN, the output is a 28 × 28 feature. Then it is flattened into a one-dimensional vector with a size of 784, which is then input into the fully connected layer. As both datasets contain 10 classes, the number of neurons in the fully connected layer is set to 10 to match the simulation task.

### Training and testing

The MNIST and Fashion-MNIST datasets are utilized for training and testing. During training, the aim is to learn the content of the two linking matrices *M* and *W*. Specifically, in this study, the models for training and testing are nonidentical. During training, the best repetition of the input stimulus is set as *n*. The model is only allowed to learn the situation where the stimulus is repeated *n* times. *n* is set to 4 in this research. The CCNN layer accepts the input signal, repeats it *n* times, and then inputs it into the fully connected layer. Finally, the output of the fully connected layer gets the classification probability of the training object through the activation function. The network adjusts the values of the matrices *M* and *W*, as well as the weight in the fully connected layer.

During testing, it is necessary to evaluate the recognition performance of each model time. When testing, the CCNN Module is repeated 100 times, and the output from each model time is fed into the fully connected layer for classification. The main difference is that for the output of the CCNN network, the training network only requires the *n*_*th*_ model time’s output as the target, whereas in the testing network, the output results of each model time need to be classified and counted^67^. Thus, the output of the CCNN network needs to be transmitted to the fully connected layer for classification after each model time. The loss function is the cross-entropy loss of the model’s predicted results and the image’s label.

For the simulation of semantic satiation caused by the same or similar repeated stimulus, the same training results are used as model parameters. The primary difference lies in whether the inputs are changed after 5 or 10 repetitions. In the visualization part, the output *Y* of each model time is converted into a grayscale image and displayed for observation. Additionally, the concepts of intra-class distance and inter-class distance are used to measure the division of semantics in the brain. The distance of the same semantics is represented by the intra-class distance. The inter-class distance is utilized to measure the difference between different semantics. Both are measured using the Euclidean distance.

The model is trained with a batch size of 200 and Adam with a learning rate of 0.001. The maximum number of epochs is set to 100. The model is implemented by Pytorch and trained on a NVIDIA GeForce GTX 1660.

### Reporting summary

Further information on research design is available in the Nature Portfolio Reporting Summary linked to this article.

### Supplementary information

Supplementary Information

Description of Additional Supplementary Files

Supplementary Data

Reporting Summary

## Data Availability

Publicly available datasets were used in this study. These datasets can be found at the following sites: http://yann.lecun.com/exdb/mnist/(MNIST), http://fashion-mnist.s3-website.eu-central-1.amazonaws.com/(Fashion MNIST). The source data for all figures of fluorescence is available in Supplementary Data. All figures are open on figshare (10.6084/m9.figshare.25507406.v2). Any further data not included in the text will be made available upon request.

## Code availability

The code used in this study is available at https://zenodo.org/records/10894595.
