# Supplementary material for: Revealing the mechanisms of semantic satiation with deep learning models
Source: Commun Biol. 2024 Apr 22;7:487. doi: 10.1038/s42003-024-06162-0 (PMC11035687; doi:10.1038/s42003-024-06162-0)
Supplement: Supplementary file 1 — Supplementary Information [file 42003_2024_6162_MOESM1_ESM.pdf]

Supplementary Information

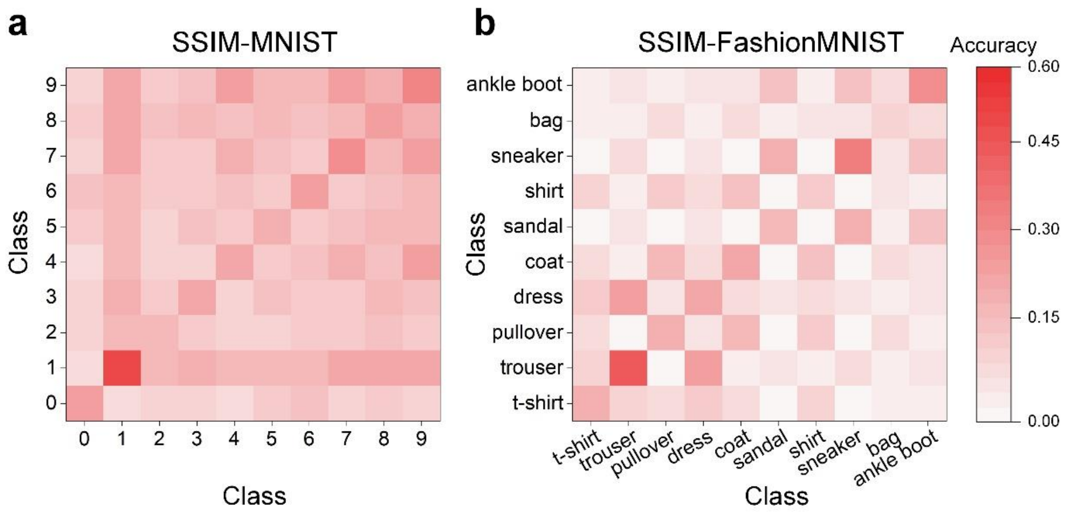

Fig. S1 Structural Similarity Index (SSIM) values. **a** SSIM of MNIST dataset. **b** SSIM of Fashion-MNIST dataset.

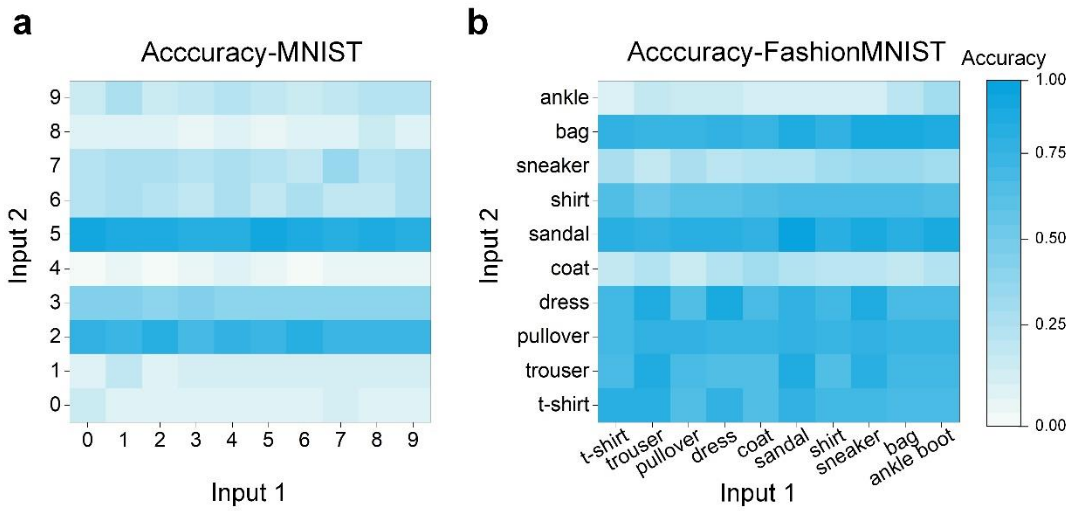

Fig. S2 The accuracy of different input1 and input2. **a** The accuracy of different input1 and input2 of MNIST dataset. **b** The accuracy of different input1 and input2 of Fashion-MNIST dataset. The sequence of results is predominantly influenced by the characteristics of input2.

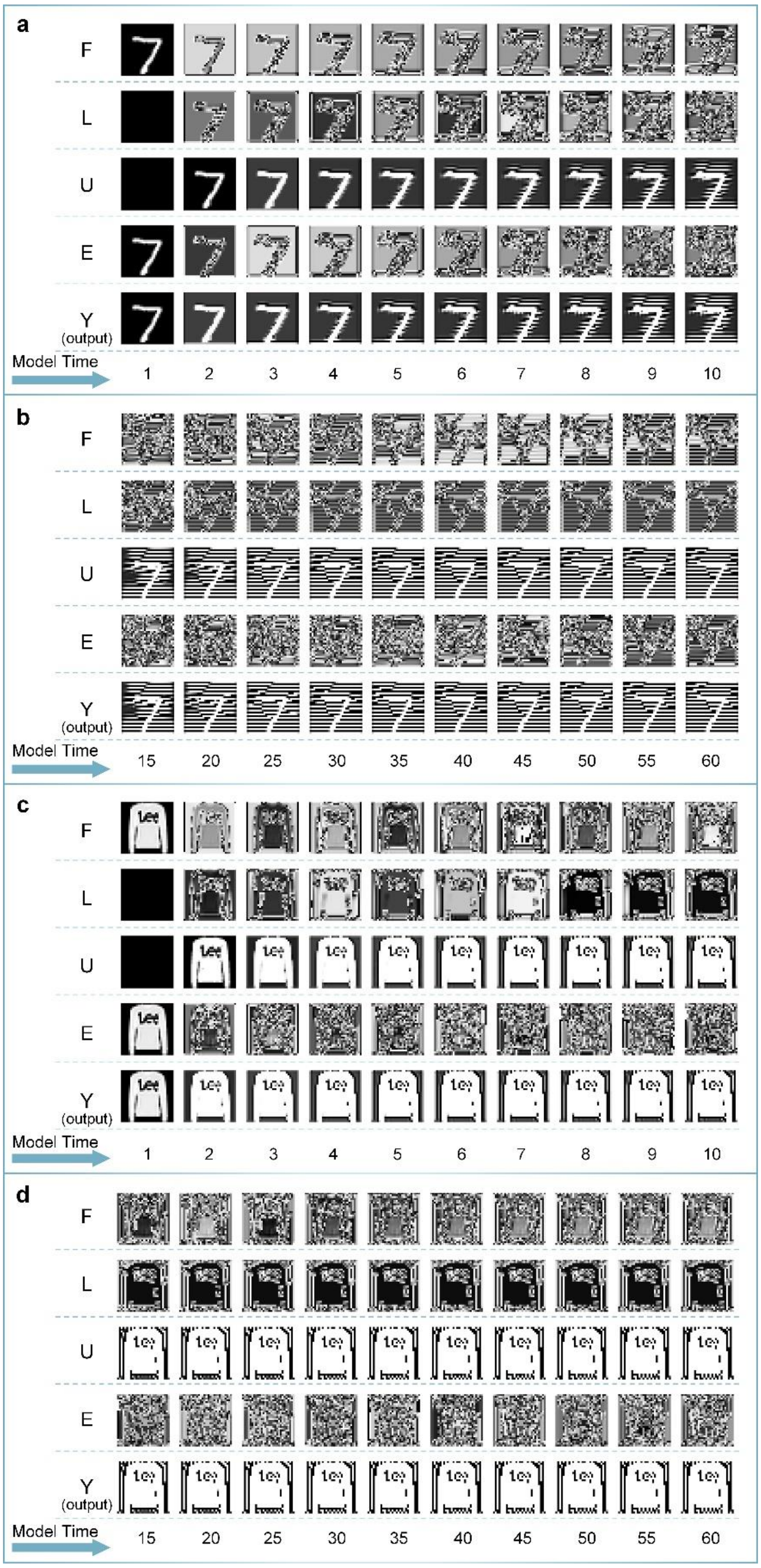

Fig. S3 Visualization of CCNN intermediate variables in semantic satiation caused by the same stimulus. **a** Visualization of changes in five variables over time for number ``7" (Model Time: 1-10). **b** Visualization of changes in five variables over time for number ``7" (Model Time: 15-60). **c** Visualization of changes in five variables over time for ``pullover" (Model Time: 1-10). **d** Visualization of changes in five variables over time for ``pullover" (Model Time: 15-60). Due to the automatic wave effect caused by the coupling connection, the noise gradually spreads to the surroundings when the time is too long, resulting in a decrease in accuracy.

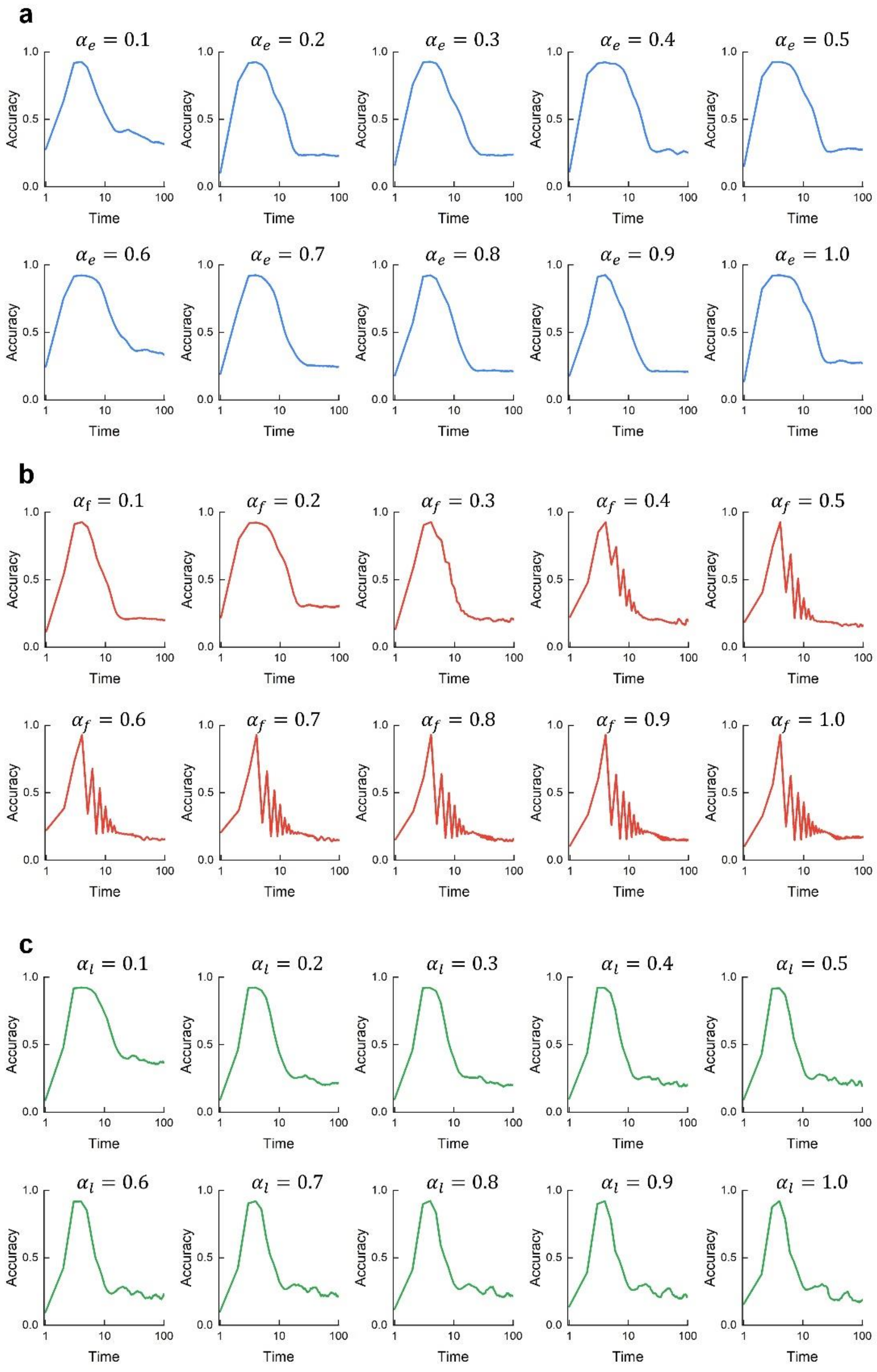

Fig. S4 The influence of attenuation coefficient α_e, α_f and α_l on results. **a** The influence of attenuation coefficient α_e on results. **b** The influence of attenuation coefficient α_f on results. **c** The influence of attenuation coefficient α_l on results. In the original experiment, parameters were set based on experience: $\alpha_f = 0.1$, $\alpha_l = 0.1$, $\alpha_e = 1$. In the subsequent analysis, everything else was kept unchanged, and only the value of a single attenuation coefficient was altered during training and testing. the influence of α_e on accuracy changes was not significant. An increase in α_f led to oscillations in the curve, while α_l caused slight variations in the accuracy level. Traditionally, a set of requirements for parameter settings suggested $\alpha_f < \alpha_e < \alpha_l$. Changes in parameters may have disrupted this pattern, impacting the model's performance.
